# Mandibular Flexure and Peri-Implant Bone Stress Distribution on an Implant-Supported Fixed Full-Arch Mandibular Prosthesis: 3D Finite Element Analysis

**DOI:** 10.1155/2018/8241313

**Published:** 2018-04-01

**Authors:** Elena Martin-Fernandez, Ignacio Gonzalez-Gonzalez, Hector deLlanos-Lanchares, Mario Andres Mauvezin-Quevedo, Aritza Brizuela-Velasco, Angel Alvarez-Arenal

**Affiliations:** ^1^Department of Prosthodontics and Occlusion, School of Dentistry, University of Oviedo, Oviedo, Spain; ^2^Department of Oral Stomatology I, Faculty of Medicine and Dentistry, University of the Basque Country, Leioa, Spain

## Abstract

**Purpose:**

The purpose of this study was to evaluate and compare the effect of three mandibular full-arch superstructures on the peri-implant bone stress distribution during mandibular flexure caused by mid-opening (27 mm) and protrusion mandibular movements.

**Materials and Methods:**

Three-dimensional finite element models were created simulating six osseointegrated implants in the jawbone. One model simulated a 1-piece framework and the other simulated 2-piece and 3-piece frameworks. Muscle forces with definite direction and magnitude were exerted over areas of attachment to simulate multiple force vectors of masticatory muscles during mandibular protrusion and opening.

**Results:**

During the movement of 27.5 mm jaw opening, the 1-piece and 3-piece superstructures showed the lowest values of bone stress around the mesial implants, gradually increasing towards the distal position. During the protrusion movement, bone stress increased compared to opening for any implant situation and for a divided or undivided framework. The 3-piece framework showed the highest values of peri-implant bone stress, regardless of the implant situation.

**Conclusions:**

The undivided framework provides the best biomechanical environment during mandibular protrusion and opening. Protrusion movement increases the peri-implant bone stress. The most mesial implants have the lowest biomechanical risk.

## 1. Introduction

Mandibular flexure is a complex mandible deformation process that changes the shape and decreases the width of the mandible arch during opening and protrusion mandibular movements due to contraction of the lateral pterygoid muscles and other masticatory muscles. Four widely recognized deformation patterns [1] have been proposed: symphyseal bending associated with medial convergence, dorsoventral shear, corporal rotation, and anteroposterior shear. Any of these deformation patterns can cause compressive, tensile, or shear stresses on the mandibular bone tissue, the range and distribution of these stresses depending on the nature and amount of force exerted by masticatory muscles, mandibular geometry, and bone quantity and quality [2]. In individuals with natural teeth and without prosthetic restorations, these stress/strain values are in line with Frost's mechanostat theory [3, 4] in the physiological adapted window or mild overload (1,500–3,000 microstrains). The periodontal ligament, which allows physiological tooth mobility, is the main factor involved in preventing an increase of stress/strain and bone loss around teeth due to mandibular flexure during functional movements or other types of movements of the mandible.

In a completely edentulous mandible restored with a conventional fixed prosthesis or an implant-supported prosthesis, a rigid structure is created, which splints two or more implants in one single unit. With this, not only is splinted teeth mobility reduced and thus the protective effect of the periodontal ligament cancelled, but also the direction of movement of the teeth changes after the splint is performed. This causes a leverage effect and different flexion forces that increase or modify bone stress/strain distribution around teeth or implants [2] in mandibular flexure during mandibular movements with or without occlusal load [2, 5–7]. Furthermore, in implant prosthodontics for an edentulous mandible, the framework design of the fixed full-arch implant-supported prosthesis affects mandibular flexure and peri-implant bone stress distribution. The framework may be designed in one piece or in two or three separate pieces. By using the 1-piece superstructure, although this design aims at evenly distributing stress among splinted implants, mandibular flexure is not counteracted, thus creating complex bending moments that increase bone stress around implants [6–9]. As some studies [10, 11] have reported that the framework can counteract mandibular flexure, this matter could be a subject for discussion. While the use of sectional prosthesis designs in 2- or 3-piece superstructures through the symphysis region has been suggested to minimize the effect of mandibular flexure and peri-implant bone stress [10, 12–14], other studies have found smaller stress values for 1-piece superstructures compared to sectioned ones [15].

With any of these framework designs, in order to have mandibular flexure, the activation of masticatory muscle contraction during mastication, grinding, and clenching or during mandibular movements without dental occlusion is necessary. In this regard, different studies have described the relationship between mandibular flexure and opening and between protrusion and lateral mandibular movements [14, 16–21], and some of them show that the protrusion movement produces the greatest mandibular deformity [17, 20, 21]. However, which combination of mandibular movements without occlusal contact and framework design of full-arch mandibular restoration promotes the best biomechanical environment is currently unclear and requires further clarification. The null hypothesis states that a single full-arch mandibular prosthesis increases peri-implant bone stress when compared to 2- or 3-piece frameworks in any mandibular movement. The objective of this paper is to compare bone stress distribution around implants of fixed full-arch mandibular restorations with 1-piece frameworks versus 2- and 3-piece frameworks subject to opening and protrusion mandibular movements.

## 2. Materials and Methods

### 2.1. Finite Element Model Design

In order to assess stress distribution in the peri-implant bone, three 3D finite element models were created. Each model was manufactured according to the shape and geometry of a completely edentulous mandible with the following dimensions: intercondylar distance of 108 mm, symphyseal height of 32 mm, chin to mandibular angle distance of 71 mm, and mandibular angle to coronoid process distance of 67 mm. Bone dimensions were 23 mm for inferosuperior height and 12 mm for buccolingual width of cortical and trabecular bone of type 2 quality, in accordance with Lekholm and Zarb classification [22]. Six implants were placed in each model in the canine and first premolar position and in the molar region at 13.5 mm, 20.5 mm, and 38.5 mm from midline, respectively. The implant Standard Plus ITI-Straumann (Institut Straumann AG, Waldenburg, Switzerland) (4.1 mm diameter, 10 mm height, and titanium alloy (Ti6Al4V)) was used as a reference for the modelling. Six solid Ti abutments (Straumann AG, Waldenburg, Switzerland), each 7 mm in height, were modelled and screwed to the implants in order to support the prosthesis framework. This feldspathic porcelain-veneered metal framework (1 mm occlusal thickness) was a superstructure made of a cobalt-chromium alloy with six retainers to be cemented. One finite element model simulated a 1-piece framework by splinting the 6 implants and the second model simulated a 2-piece framework divided along the midline, while the third model simulated a 3-piece framework with two posterior sections and an anterior section.

### 2.2. Material Properties and Interface Conditions

All the materials used in these models are considered to be linearly elastic, homogeneous, and isotropic. The values of Young's moduli and Poisson's ratio values were taken from published data [8, 23–26] (Table 1). The bone-implant interface was considered to be perfect, with 100% osseointegration, and the passive fit between the abutments and the superstructure was also considered to be perfect. The cement layer between abutments and the framework retainers was not taken into consideration; those structures were assumed to be completely bonded without any loosening and the same is true between the framework and veneering material (feldspathic porcelain).

### 2.3. Loading and Boundary Conditions

Mid-opening (27 mm) and protrusion mandibular movements were simulated. In order to recreate movements, the model was loaded with groups of force vectors recreating the action of muscles on each side (anterior, middle, and posterior temporalis; superficial and deep masseter; medial pterygoid; and superior and inferior lateral pterygoid). The direction and degree of muscular force vectors were applied in accordance with previous studies [6, 10, 12, 13, 27, 28]. The individual forces of each muscle were determined taking two assumptions into consideration: firstly, the force applied by each muscle is proportional to the product of its cross-sectional area (*X*_mi_) and a constant force per unit of the muscular cross-sectional area (*K*), and, secondly, mandibular movements imply a certain amount of muscular activation which will depend upon the phase of the muscle and the type of movement the muscle is performing. Thus, a muscle can be at 100% of its activity during a particular movement and at just 50% when performing another type of movement. Consequently, the resultant vector of muscle force (*M*_ir_) for a particular muscle in isometric contraction during a specific movement could be given by the product [*X*_mi_ · *K*] × EMG_mi_ = *M*_ir_, where EMG_mi_ is the value of the muscle contraction relative to its maximum response for each type of specific movement; and the product [*X*_mi_ · *K*] is the weighting factor assigned to each muscle (Table 2). Therefore, the components of the orthogonal force vector are determined by multiplying *M*_ir_ by its corresponding unit vector [28]. Subsequently, these orthogonal force vectors provide the nodes forming the corresponding area of muscular attachment. Table 2 shows the forces and force vectors applied to simulate mid-opening (27 mm) and protrusion mandibular movements.

Furthermore, three-dimensional restraints were placed bilaterally, acting perpendicularly to the occlusal plane and allowing freedom of displacement only in the horizontal plane. Restraints were also placed bilaterally on the condyles, so both of them could rotate around a transversal axis passing through the condylar medial poles but without any displacement taking place [12, 28].

The finite element models were created and meshed using ANSYS 11.0, commercial 3D finite element software (Ansys, Inc., Canonsburg, PA). To generate meshes, a 10-node quadratic tetrahedral element with 3 degrees of freedom per node was used. The model simulating the 1-piece framework was composed of 60,097 nodes and 65,872 solid elements; the 2-piece framework model consisted of 62,362 nodes and 73,328 solid elements, and, finally, the 3-piece framework was composed of 88,603 nodes and 80,517 solid elements.

## 3. Results

Table 3 illustrates the stress on peri-implant bone and implants for the three models. During mandibular mid-opening movements, the smallest bone stress occurred around mesial implants at both sides, progressively increasing towards more distal positions, except for the 3-piece framework prosthesis, where the highest peri-implant bone stress was recorded in implants in the first premolar location. For these implants and for those at the distal end, the 2-piece framework showed higher bone stress values when compared with the 1- and the 3-piece frameworks.

During mandibular protrusion movements, in any of the implant positions and with all types of framework, stress values considerably increased but not evenly as in the opening movements. Regardless of the type of framework, the smallest bone stress values were recorded around mesial implants. The peri-implant bone stress values increased by a factor of at least two in intermediate positioned implants when compared to those at mesial position and decreased in implants at distal position but with values even greater than those recorded at mesial implants (see Table 2). In any event, the 3-piece framework provided the worst biomechanical environment, yielding the highest values of peri-implant bone stress at any implant position compared to the 1- or 2-piece frameworks.

**Figure 1:**
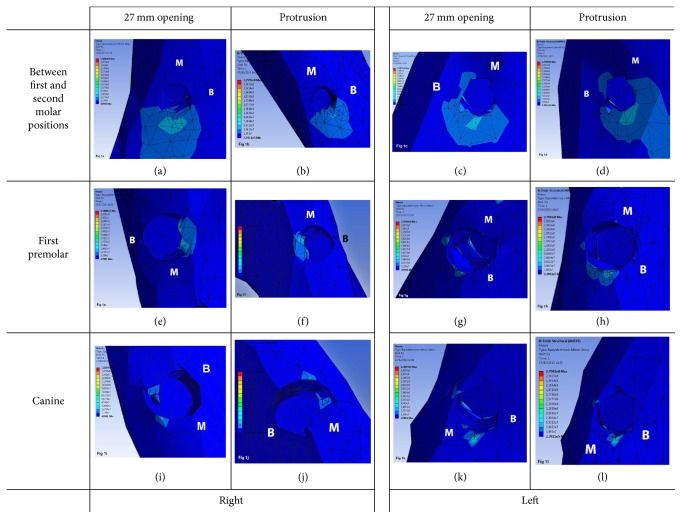
Distribution and location of stress in peri-implant bone in 1-piece framework during 27 mm opening and protrusion (B: buccal; M: mesial surfaces).* 1-piece framework images*: (a) right side: between first and second molar positions during 27 mm opening; (b) right side: between first and second molar positions during protrusion; (c) left side: between first and second molar positions during 27 mm opening; (d) left side: between first and second molar positions during protrusion; (e) right side: first premolar position during 27 mm opening; (f) right side: first premolar position during protrusion; (g) left side: first premolar position during 27 mm opening; (h) left side: first premolar position during protrusion; (i) right side: canine position during 27 mm opening; (j) right side: canine position during protrusion; (k) left side: canine position during 27 mm opening; (l) left side: canine position during protrusion.

**Figure 2:**
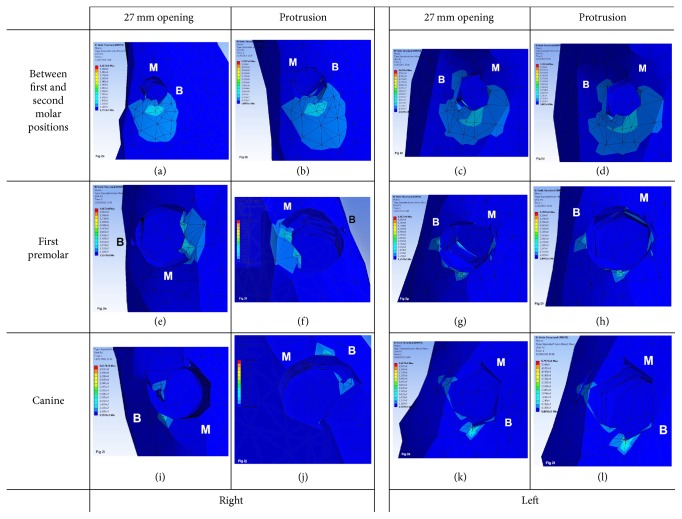
Distribution and location of stress on peri-implant bone in 2-piece framework during 27 mm opening and protrusion (B: buccal surface; M: mesial surface).* 2-piece framework images*: (a) right side: between first and second molar positions during 27 mm opening; (b) right side: between first and second molar positions during protrusion; (c) left side: between first and second molar positions during 27 mm opening; (d) left side: between first and second molar positions during protrusion; (e) right side: first premolar position during 27 mm opening; (f) right side: first premolar position during protrusion; (g) left side: first premolar position during 27 mm opening; (h) left side: first premolar position during protrusion; (i) right side: canine position during 27 mm opening; (j) right side: canine position during protrusion; (k) left side: canine position during 27 mm opening; (l) left side: canine position during protrusion.

**Figure 3:**
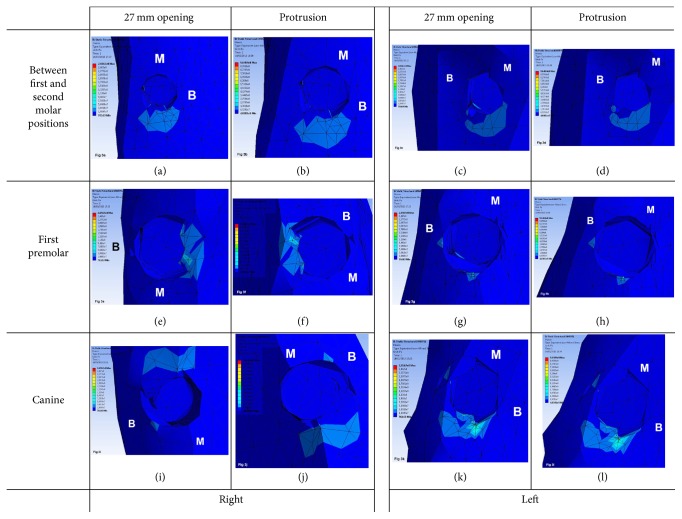
Distribution and location of stress on peri-implant bone in 3-piece framework during 27 mm opening and protrusion (B: buccal; M: mesial surfaces).* 3-piece framework images*: (a) right side: between first and second molar positions during 27 mm opening; (b) right side: between first and second molar positions during protrusion; (c) left side: between first and second molar positions during 27 mm opening; (d) left side: between first and second molar positions during protrusion; (e) right side: first premolar position during 27 mm opening; (f) right side: first premolar position during protrusion; (g) left side: first premolar position during 27 mm opening; (h) left side: first premolar position during protrusion; (i) right side: canine position during 27 mm opening; (j) right side: canine position during protrusion; (k) left side: canine position during 27 mm opening; (l) left side: canine position during protrusion.

As for the restoration that splints all implants in a single framework and irrespective of the type of mandibular movement, peri-implant bone stress in distal implants was located in the distal area, dissipating towards the lingual, vestibular, and distal region and vertically down to the first 2-3 threads of the implant. However, in implants at an intermediate position, bone stress was in the lingual and distal region of the implant on the left side and only in the lingual region on the right side. In mesial implants, bone stress was located in small mesiolingual and distolingual regions of the right implant and in the mesial region of the left implant exhibiting stress in the linguoapical direction (Figure 1). In the framework model separated at midline, the distribution and location of bone stress on distal and intermediate implants are similar to what has been described for the 1-piece framework. However, in mesial implants, the distribution of bone stress is different, being located in the vestibular region on the right side and in the distal region on the left side (Figure 2). Likewise, such a bone stress trend is also observed for the 3-piece framework, except for the mesial implants that exhibit in the distal region a bigger stress concentration and dissipation surface, mainly on the left side (Figure 3).

## 4. Discussion

### 4.1. Clinical and Biological Implications

This study establishes that the framework in a single piece exhibits a better biomechanical environment with smaller bone peri-implant stress values for all implant locations. By contrast, in the case of the 3-piece framework, higher bone stress values occurred around implants in protrusion mandibular movements. This result supports the theory that rigid splinting of the full mandibular arch can provide additional resistance, thus counteracting the effects of mandibular flexure when there is a single unilateral posterior framework [15]. This agrees with other research studies that, by simulating occlusion at maximum intercuspation, report that unseparated superstructures are more effective in relieving peri-implant bone stress compared to the separated ones [15]. In contrast, other previous studies have shown smaller stress values and a greater inhibition of mandibular deformities with 3-piece superstructures versus those of 2 pieces [12] and also smaller stress values in superstructures separated at midline compared to unseparated frameworks [10].

In this study, the protrusion movement exhibited the greatest mandibular flexure effect, according to clinical and biomechanical trials describing greater mandibular deformity and stress/strain during protrusion movements than opening or lateral mandibular movements [20, 28, 29]. The results could be explained through the differences at the beginning of activity of the lateral pterygoid muscles compared to opening movements together with previous study findings that describe the existence of greater medial convergence and corporal approximation during protrusion [20, 28, 29]. This could be clinically relevant in clenching parafunctional habits with incisal edge-edge or beyond the incisal edge contact positions and less relevant in normal mastication, where wide protrusion movements are not usual. Nonetheless, during protrusion, the results show that the greatest risk of peri-implant bone loss is found in an intermediate or posterior position on both sides with a separated framework of 2 and 3 pieces. This is in accordance with some of the similar studies with a 1-piece framework [10]. On the other hand, it disagrees with studies of superstructures separated at midline [10] and with most of the research studies with or without interforaminal implants, describing higher stress values in the more mesial sections [6, 10, 13], decreasing towards implants more distally placed [5, 12, 13].

According to the present study's data, the lowest mandibular flexure and peri-implant bone stress was recorded in the opening mandibular movement at 27 mm in premolar peri-implant bone with the 2-piece framework. This result supports the relationship between mandibular flexure and mouth opening, both in clinical [14, 16, 20, 21, 29] and biomechanical studies [10, 28]. Furthermore, it is in line with studies describing mandibular flexure from the beginning of the mouth opening movement with simultaneous medial convergence, corporal rotation, and dorsoventral shear [14, 20, 29]. Mid-opening (27 mm) of the mouth hardly modifies the distribution of the peri-implant bone stress when compared to protrusion. By contrast, the lowest stress values were recorded in the mesial implants closest to the mandibular symphysis. This result contradicts the symphyseal bending and the high strain in the symphysis of an adult* Macaca fascicularis* during mouth opening [1], as well as later surveys describing similar data [10, 12, 13], although some in vivo studies may support the existence of a greater stress on posterior implants, because at maximum opening the mandibular arch is reduced at the site of the second molars more than at the site of the canines [16]. The explanation could be linked to the shape and dimensions conferred to the symphyseal area in the finite element analysis, because in vivo surveys confirm that the higher values of mandibular deformation occurred in subjects with lower symphysis height [5, 7, 19], with a contralateral counteraction in 1-piece frameworks, as it has been suggested that cross-arch prostheses significantly restrict flexure of the mandible [10]. However, another possible explanation might be an interruption or cancellation of the mandibular flexure effect in the 2- or 3-piece superstructures, because as there are different mandibular deformation patterns, it seems reasonable to accept that a given framework could affect each deformation pattern differently. However, the small peri-implant bone stress values recorded during opening movements do not support the idea that, during the normal masticatory function, where opening and closing mouth movements occur continuously, there may be a greater risk of bone loss around implants, even in patients with a full-arch prosthesis separated at midline.

The finding of bone stress around the neck and first threads of the implant, irrespective of mandibular movement and the type of framework, is in agreement with numerous different biomechanical studies with or without mandibular flexure [10, 12, 15] and has also been supported by clinical studies. However, mandibular flexure qualitatively modifies bone stress in this area depending on deformation patterns. In this study, a dorsoventral shear deformation pattern combined with a corporal rotation pattern or other patterns could explain why stress is more frequently located in distal and mesial peri-implant bone regions, except for intermediate implants where bone stress occurs lingually. In other studies, buccal and lingual areas were the more common sites for bone stress localization [10, 12, 13].

### 4.2. Limitations and Justification of the Finite Element Design

The 3D finite element analysis method has been widely used in dental literature to evidence the stress/strain in the peri-implant bone, implant/abutment complex, and superstructures in many different situations. However, it is not possible for a mathematical/computational model to reproduce as exactly as possible all biological characteristics and, therefore, the FEA must assume simplifications with respect to properties of materials, loading, and boundary conditions. Consequently, the results obtained do not exactly correspond to the results obtained in clinical practice, being only an approach to the clinical situation. In this study, it is assumed that all materials and volumes are homogeneous and have a linear elastic isotropic behaviour. This is also a limitation and it should be taken into consideration in order to improve the accuracy of the estimates. Furthermore, it is also assumed that all interfaces are continuous and that 100% osseointegration is achieved, which is also a limitation. However, some FEA studies have closely recreated mandibular flexure patterns observed in vivo [20, 28]. During mandibular movements, condyles rotate and can also be displaced, though they were fixed on both sides as in other studies [10, 12, 13, 28, 30]. Boundary conditions, ligaments, and temporomandibular joint structures were not considered either. Furthermore, it is not possible to exactly simulate muscular and activity patterns. Considering the aforementioned simplifications and assumptions, the distribution data of this study are to be understood in qualitative terms rather than in quantitative terms.

Within the limitations of this 3D finite element analysis and in accordance with the data obtained, the following* conclusions* may be drawn:

(1) The division of the framework and type of mandibular movement influence mandibular flexure and peri-implant bone stress.

(2) The mandibular flexure, which occurs during protrusion movement, shows the highest values of bone stress in the three types of framework.

(3) The undivided framework shows a mandibular flexure with the least peri-implant bone stress regardless of mandibular movement.

(4) The null hypothesis is rejected.

## Figures and Tables

**Table 1:** Mechanical properties of modelled materials and structures used for three-dimensional finite analysis.

Material	Structure	Young's modulus (GPA)	Poisson's ratio	References
Titanium	Implant	110.0	0.35	Sevimay et al., 2005 [23]
Titanium alloy	Abutment	107.2	0.33	Suansuwan and Swain, 2001 [24]
Cr-Co alloy	Framework prosthesis	218.0	0.33	Anusavice and Coscone, 2003 [25]
Feldspathic porcelain	Veneered framework prosthesis	68.9	0.28	Geng et al., 2001 [8]
Cortical bone	Peri-implant bone	13.70	0.30	Borchers and Reichart, 1983 [26]
Cancellous bone	Peri-implant bone	1.37	0.31	Borchers and Reichart, 1983 [26]

**Table 2:** Number and magnitudes of muscle loads used in the finite element analysis model during opening (27 mm) and protrusion mandibular movements. The *x*, *y*, and *z* coordinates represent the muscle loads in Newton in each direction. All coordinates are referenced to a global Cartesian coordinate system (*x*-*y* = frontal plane, *x*-*z* = horizontal plane, and *y*-*z* = anteroposterior plane).

Muscles	Group cross section (cm^2^)	Muscle weighting factor (N)	27 mm opening						Protrusion					
			Right			Left			Right			Left		
			*x*	*y*	*z*	*x*	*y*	*z*	*x*	*y*	*z*	*x*	*y*	*z*
Superior masseter	2.38	190.4	−64.736	274.18	131.176	−64.736	274.176	131.376	−97.104	407.456	194.208	−97.104	407.456	194.208
Deep masseter	1.02	81.6	−12.24	17.136	−8.16	−12.24	17.136	−8.16	−15.504	20.4	−9.792	−15.504	20.4	−11.424
Medial pterygoid	1.9	174.8	120.612	195.78	92.644	115.368	187.036	87.4	311.144	505.92	239.476	297.16	484.196	227.24
Anterior temporalis	2.4	158.0	−744.18	492.96	1.58	−744.18	492.96	1.58	−1273.5	837.4	37.92	−1273.48	837.4	37.92
Medial temporalis	1.1	95.6	−23.9	86.996	−52.58	−28.68	104.204	−62.14	−4.78	16.252	−9.56	−7.648	26.768	−16.252
Posterior temporalis	0.7	75.6	−21.168	47.628	85.428	−21.168	47.628	85.428	−3.78	9.072	15.876	−3.024	6.804	10.584
Inferior lateral pterygoid	1.1	66.9	421.47	−117.1	508.44	421.47	−117.075	508.44	418.125	−115.74	502.419	418.125	−115.74	502.419
Superior lateral pterygoid	1.0	28.7	122.262	11.767	103.607	119.105	11.48	101.024	133.168	12.915	112.791	133.168	12.915	112.791

**Table 3:** Maximum von Mises stress values (MPa) in peri-implant bone and implants of 1-, 2-, and 3-piece frameworks during mid-opening (27 mm) and protrusion mandibular movements.

Implant's location	1-piece framework		2-piece framework		3-piece framework	
	Protrusion	Opening	Protrusion	Opening	Protrusion	Opening
Between right first and second molar positions	60.12	40.22	130.29	60.27	120.39	70.28
	(742)	(425)	(1850)	(522)	(1620)	(459)
Right first premolar	90.26	34.48	270.34	40.54	200.19	80.36
	(775)	(391)	(1690)	(470)	(1490)	(417)
Right canine	50.17	30.61	113.20	30.15	80.69	30.47
	(799)	(543)	(2140)	(600)	(2200)	(616)
Left canine	38.75	30.59	113.31	30.02	80.43	30.09
	(986)	(493)	(1920)	(535)	(1760)	(490)
Left first premolar	75.36	34.39	270.43	40.78	200.37	80.58
	(883)	(438)	(1830)	(513)	(1630)	(453)
Between left first and second molar positions	60.15	40.19	130.39	60.43	120.41	70.67
	(808)	(458)	(1900)	(545)	(1670)	(480)
